# Wolf genetic diversity compared across Europe using the yardstick method

**DOI:** 10.1038/s41598-023-40834-x

**Published:** 2023-08-22

**Authors:** Maja Jan, Astrid Vik Stronen, Barbara Boljte, Rok Černe, Đuro Huber, Ruben Iosif, Franc Kljun, Marjeta Konec, Ivan Kos, Miha Krofel, Josip Kusak, Roman Luštrik, Aleksandra Majić Skrbinšek, Barbara Promberger–Füerpass, Hubert Potočnik, Robin Rigg, Peter Trontelj, Tomaž Skrbinšek

**Affiliations:** 1 Biotechnical Faculty, University of Ljubljana, Jamnikarjeva 101, 1000 Ljubljana, Slovenia; 2 DivjaLabs d.o.o., Aljaževa ulica 35a, 1000 Ljubljana, Slovenia; 3 Department of Chemistry and Bioscience, Aalborg University, Fredrik Bajers Vej 7H, 9220 Aalborg, Denmark; 4 Slovenia Forest Service, Večna pot 2, 1000 Ljubljana, Slovenia; 5 Faculty of Veterinary Medicine, University of Zagreb, Vjekoslava Heinzelova 55, 10000 Zagreb, Croatia; 6 Foundation Conservation Carpathia, 27 Calea Feldioarei, 500471 Brașov, Romania; 7 Genialis Inc, Vojkova cesta 63, 1000 Ljubljana, Slovenia; 8 Slovak Wildlife Society, Belanská 574/6, P.O. Box 72, Liptovský Hrádok, 033 01 Slovakia

**Keywords:** Evolution, Molecular biology, Genetics, Genetic markers, Population genetics

## Abstract

Integrating data across studies with traditional microsatellite genetic markers requires careful calibration and represents an obstacle for investigation of wide-ranging species where populations require transboundary management. We used the “yardstick” method to compare results published across Europe since 2002 and new wolf (*Canis lupus*) genetic profiles from the Carpathian Mountains in Central Europe and the Dinaric Mountains in Southeastern Europe, with the latter as our reference population. We compared each population with Dinaric wolves, considering only shared markers (range 4–17). For each population, we calculated standard genetic diversity indices plus calibrated heterozygosity (*Hec*) and allelic richness (*Ac*). *Hec* and *Ac* in Dinaric (0.704 and 9.394) and Carpathian wolves (0.695 and 7.023) were comparable to those observed in other large and mid-sized European populations, but smaller than those of northeastern Europe. Major discrepancies in marker choices among some studies made comparisons more difficult. However, the yardstick method, including the new measures of *Hec* and *Ac*, provided a direct comparison of genetic diversity values among wolf populations and an intuitive interpretation of the results. The yardstick method thus permitted the integration of diverse sources of publicly available microsatellite data for spatiotemporal genetic monitoring of evolutionary potential.

## Introduction

Comparison of results from traditional microsatellite genetic markers across studies requires calibration and thus complicates broad-scale studies^1^. This has negative consequences for evolutionary research and conservation management of wide-ranging species where many populations range across multiple international boundaries^2–4^ and single studies rarely include entire populations. Rapid environmental changes and associated range shifts in many wild species further augment the need to understand their population genetic structure and evolutionary potential across wider scales (e.g., Ref.^5^). New genomic techniques are increasingly permitting broad-scale investigation also for non-model organisms, including marine and terrestrial taxa (e.g., Ref.^6,7^). However, many genetic monitoring programs still use microsatellite markers, which offer important temporal perspectives in cases where DNA sources no longer exist, but legacy data are available in the form of microsatellite profiles^8^. Given the comparative strength of microsatellite markers for detecting changes across limited temporal and spatial scales^9^, more efforts are needed to mitigate the problems that researchers and conservation managers encounter when seeking to integrate these valuable datasets. Efforts toward standardizing non-invasive genetic methods would also greatly facilitate the study of dispersal, and in turn help discern biological differences in dispersal parameters from variability owing to differences in methodology (reviewed in Ref.^10^).

To help alleviate these constraints, a “yardstick” method was proposed for comparison of genetic diversity indices among populations and applied to brown bears (*Ursus arctos*) as a relevant example^11^. The method calibrates genetic diversity indices to a common denominator, the reference or “yardstick” population, allowing comparisons to be made. The authors suggested that this method may be even more useful for species with more diverse (i.e., less overlapping) sets of genetic markers used in different studies, as is the case with the grey wolf (*Canis lupus*)^3^. The yardstick method could facilitate comparison of existing data from populations with diverse demographic histories^11^, including habitat fragmentation, bottlenecks, and founder effects. Notably, allelic diversity has been proposed as a better predictor and candidate proxy than heterozygosity of evolutionary potential^12^. Recent works maintain that genetic diversity is vital for evolutionary fitness (reviewed in Ref.^13^) and that within-species genetic diversity merits wider recognition in international conservation planning^14^. The yardstick method’s inclusion of allelic richness could thus provide a flexible and cost-effective measure for genetic monitoring, research, and applied conservation management.

The wolf is a wide-ranging species where discrepancies in genetic markers among studies limit comparisons at relevant spatial scales in Europe^3^. A review of wolf population genetics across Europe found genetic diversity to be high in northeastern Europe and decreasing toward the southwest^15^, and the authors reported that observed and expected heterozygosity showed a clear spatial trend whereas allelic richness did not. They also noted the potential of the yardstick method but were unable to use this approach to analyze the wolf microsatellite data across the continent because of too few (≤ 3) overlapping loci.

In recent years, new genetic data on wolves has become available, allowing comparison of findings across regional scales, based on a larger number of common markers. These new data include populations in Central and Southeastern Europe where relatively abundant and persistent populations of large carnivores^2^ are thought to represent vital genetic diversity for long-term preservation of evolutionary potential. Whereas wolves in many parts of Europe are well-studied at various spatio-temporal scales, those in Central and Southeastern Europe have received less attention (Supplemental Tables S1 and S2) and have high priority for conservation^16^.

Analyses across genetic markers—including neutral and potentially adaptive genetic variation—show that wolves from the Carpathian Mountains in Central Europe are separated from wolves farther north^17,18^, although there is evidence of recent gene flow into the Carpathians^19^. The Carpathian population spans the Czech Republic, Slovakia, Poland, Hungary, Ukraine, Serbia, and Romania and is among the largest in Europe^20^, estimated to number c. 3900–4700 individuals^16^.

Recent population genetic analyses of microsatellite markers showed spatial structure within Slovakia that may have been influenced by environmental and anthropogenic factors^19^. Wolves were almost extirpated twice from Slovakia during the twentieth century but recovered and have since become relatively abundant in the central and eastern regions^21,22^. Moreover, further research is needed to evaluate connectivity across the region. A recent genome-wide analysis found signs of weak genetic structure between wolves in Romania and the Carpathian Mountain region of Ukraine^23^. Additional analyses with microsatellite markers could help clarify the extent of genetic exchange across short spatio-temporal scales^9^ and whether there are signs of recent resistance to gene flow.

The Carpathian Mountain wolves were reported to be genetically distinct from the Dinaric-Balkan population^18,24,25^. The latter spans ten countries, from the Italian—Slovenian border in the west, across the Dinaric Mountains, to the Balkan Peninsula and Rhodope Mountains in Greece and Bulgaria in the southeast, with an estimated size of c. 5000–5500 individuals^16,26^. Wolves in the northwestern Dinaric Mountains (NW Dinaric) were subject to severe persecution in the past and were almost extirpated during the eighteenth and nineteenth centuries. The population started to recover in the second half of the twentieth century^27,28^ and is currently expanding its range into the Alps, where contact with the Alpine population has already been established^29,30^. Earlier findings from Croatia and Bosnia & Herzegovina have reported findings of genetic structure based on microsatellite markers^29,31^ and although such differentiation was not observed in Serbia^32^, there was evidence of structure based on mitochondrial DNA^33^, suggesting the need for broader-scale analyses.

The main objectives of this study were to (1) place new findings on wolf genetic diversity in the Carpathian and Dinaric regions into a broader context at the European level using previously published studies and (2) apply the yardstick method to a model species where conservation managers require a better understanding of genetic diversity at the continental scale. We also extended the yardstick method with calculation of calibrated heterozygosity and allelic richness estimates, enhancing the interpretability of the results. Finally, we discuss the relevance of these measures for conservation management of wolves and other wide-ranging species.

## Materials and methods

### Sampling

We included 345 wolf profiles from tissue and noninvasive samples from the Dinaric Mountains (Slovenia, n = 145 and Croatia, n = 148) and the Carpathian Mountains (Romania, n = 32 and Slovakia n = 20; Supplemental Fig. S1), after removal of individuals showing any sign of possible hybridization with dogs. For details about sampling, laboratory processing, genotyping and error checking, assessment of hybridization, and identification of wolves, see Supplementary Note S1, Supplemental Tables S3, and S4. We obtained genotypes for 34 microsatellite markers for Dinaric wolves, and a subset of 21 markers for Carpathian wolves. We henceforth refer to wolves in Slovenia and the Gorski kotar region of northwestern Croatia—our reference population—as the northwestern Dinaric (NW Dinaric) population. The broader Dinaric Mountain study area also comprised the Croatian regions of Lika and Dalmatia further south (Supplemental Fig. S1).

### Estimation of genetic diversity, reference wolf population and comparison of populations

For data handling, calculation of genetic diversity indices—observed heterozygosity (*Ho*), expected heterozygosity (*He*) and allelic diversity (*A*)—and estimation of genotyping error rates, we used the package adegenet v.2.1.8^34^ in R v.4.2.2^35^. We used the R package pegas v. 1.0-1^36^ to estimate departures from Hardy–Weinberg (HW) equilibrium and corrected for multiple testing with the Holm-Bonferroni multiple test correction with alpha = 0.05^37^.

We collected published genetic diversity data based on microsatellites for other European gray wolf populations through literature review (Supplemental Tables S1 and S2). Where diversity indices were published per locus and, at times, per population (for studies that reported population genetic structure), we compared allelic richness using the yardstick reference population method^11^. For the purpose of this study, we use ‘population’ to refer to sampling areas and/or population genetic clusters reported in the original studies, although it is important to note that sampling may have been defined by national and regional jurisdictions and does not always correspond with broad-scale studies, where genome-wide analyses have found several transboundary units comprising multiple countries (e.g., Ref.^18,38,39^). We performed pairwise comparison whereby each population of interest was compared with a reference population, considering only the genetic markers shared between the two studies. Differences in sample size were corrected through resampling with replacement multiple times (~ 1000) from the larger sample size to the sample size of the smaller data set. For the resulting subsamples, genetic diversity indices with their standard errors were calculated (*A, He* and *Ho*). Heterozygosity ratio (*Her*) and allelic richness ratio (*Art*) indices were calculated with the corresponding reference population values in the denominator^11^. As an extension of the method described in the original paper, we multiplied the *Her* and *Art* ratio indices with heterozygosity or, respectively, allelic richness of the reference population, obtaining directly interpretable calibrated expected heterozygosity (*Hec*) and allelic richness (*Ac*) values. As the values of these parameters were on the same scale as the original heterozygosity and allelic richness, this allowed both direct comparison between otherwise incompatible datasets as well as a more intuitive interpretation of the obtained values. As the relationship between observed and expected heterozygosity is better estimated in the original data (more loci, no resampling), we maintained the relationship between these two parameters in each population by calculating the calibrated observed heterozygosity (*Hoc*) from *Hec* and the ratio between observed (*Ho*) and expected (*He*) heterozygosity in the original data as *Hoc* = Hec * (Ho/He).

The NW Dinaric population with a relatively large sample size and locus set was used as the reference population (Supplemental Table S2, Supplemental Fig. S1, Supplemental Note S1). To avoid a possible sampling intensity bias in Slovenia and Croatia, we randomly selected 76 out of 145 individuals from Slovenia to match the number of wolves sampled in Gorski kotar (58), considering that the estimated number of wolf packs living in each area is comparable^40^. The final reference population included 134 individuals. Analyses were performed in R using the “resamplediversity” package^11^ (also available on GitHub (https://github.com/romunov/resamplediversity) and implemented in the function divRatio of the diveRsity package^41^). For each comparison we ran 1000 random subsamples. When using the yardstick method to calculate diversity indices for populations with sample sizes larger than the reference population, we compared the published indices directly (i.e., without resampling) using the loci in common. In these cases, the number of samples was large in both populations and the relative sample sizes were very similar, and any bias should therefore be minimal. Figures were created using base R functions and the R package ggplot2^42^. Maps were created using QGIS 3.24.0^43^.

## Results

### Dinaric and Carpathian wolves

**Figure 1:**
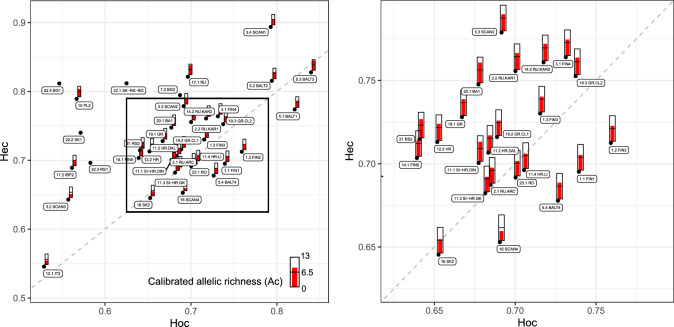
Observed (Ho) versus expected (He) heterozygosity and calibrated allelic richness (Ac) with wolves in NW Dinaric (Slovenia and Gorski kotar, Croatia) used as the reference population. Dashed diagonal line presents the Hardy–Weinberg (HW) equilibrium of Ho = He, and the positions of the points indicate the direction of deviation from HWE. The graph on the right is a close-up of the marked region in the first image. Some studies did not report values for all parameters as noted for Supplemental Table S2.

For Dinaric wolves, locus FH2004 indicated the presence of null alleles (estimated frequency 0.10) and was therefore excluded (Supplemental Note S2, Supplemental Table S3). Genetic diversity values were comparable among the NW Dinaric, Lika, Dalmatia, and Romanian populations whereas Slovakian wolves, where we had the smallest sample size, showed somewhat lower values (Table 1; Fig. 1; Supplemental Table S2).

**Table 1:** Genetic diversity measures for Dinaric (NW Dinaric, Lika, Dalmatia) and Carpathian (Slovakia and Romania) wolves.

Populations	*N*	*A* (*s.e.*)	*He* (*s.e.*)	*Ho* (*s.e.*)
NW Dinaric (Slovenia and Gorski kotar, HR)	134	6.62 (0.32)	0.673 (0.021)	0.661 (0.025)
Lika (HR)	34	5.91 (0.27)	0.687 (0.018)	0.699 (0.023)
Dalmatia (HR)	56	6.47 (0.27)	0.702 (0.016)	0.680 (0.020)
Slovakia	20	4.86 (0.22)	0.640 (0.031)	0.650 (0.039)
Romania	32	5.71 (0.33)	0.690 (0.025)	0.700 (0.039)

*N* number of individuals, *A* allelic diversity, *He* expected heterozygosity, *Ho* observed heterozygosity, with standard error (*s.e*.).

### Comparison of genetic diversity of Dinaric and Carpathian wolves with other European populations

**Figure 2:**
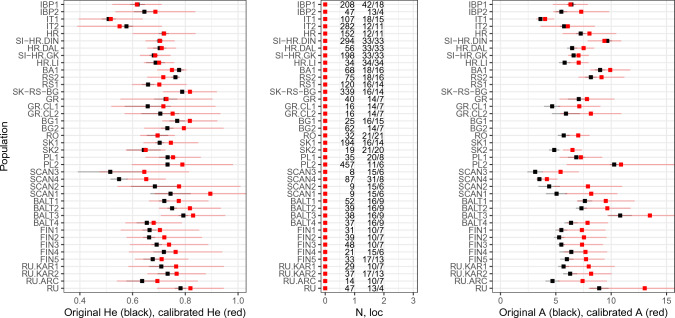
Comparison of original and calibrated heterozygosity and allelic richness values for European wolf populations. N = number of samples, loc/used = number of loci in the population/number of loci used in comparisons. Lines indicate the confidence interval of each estimate (± 1.96 × s.e.).

For the studies we examined, the number of microsatellite loci ranged from 10^44^ to 42^45^ (Supplemental Table S2; Fig. 2). The minimum number of loci shared between our reference population and other populations was four, for wolves from the Iberian Peninsula and Russia^46^. We also included data from other wolf studies from these areas that shared a higher number of loci with the reference population. For wolves from the Iberian Peninsula, we used data from Ref.^45^, and for wolves in NW Russian we used results from Ref.^47,48^. The maximum number of loci shared between our reference population and another wolf population was 17, for Iberian wolves^45^.

**Figure 3:**
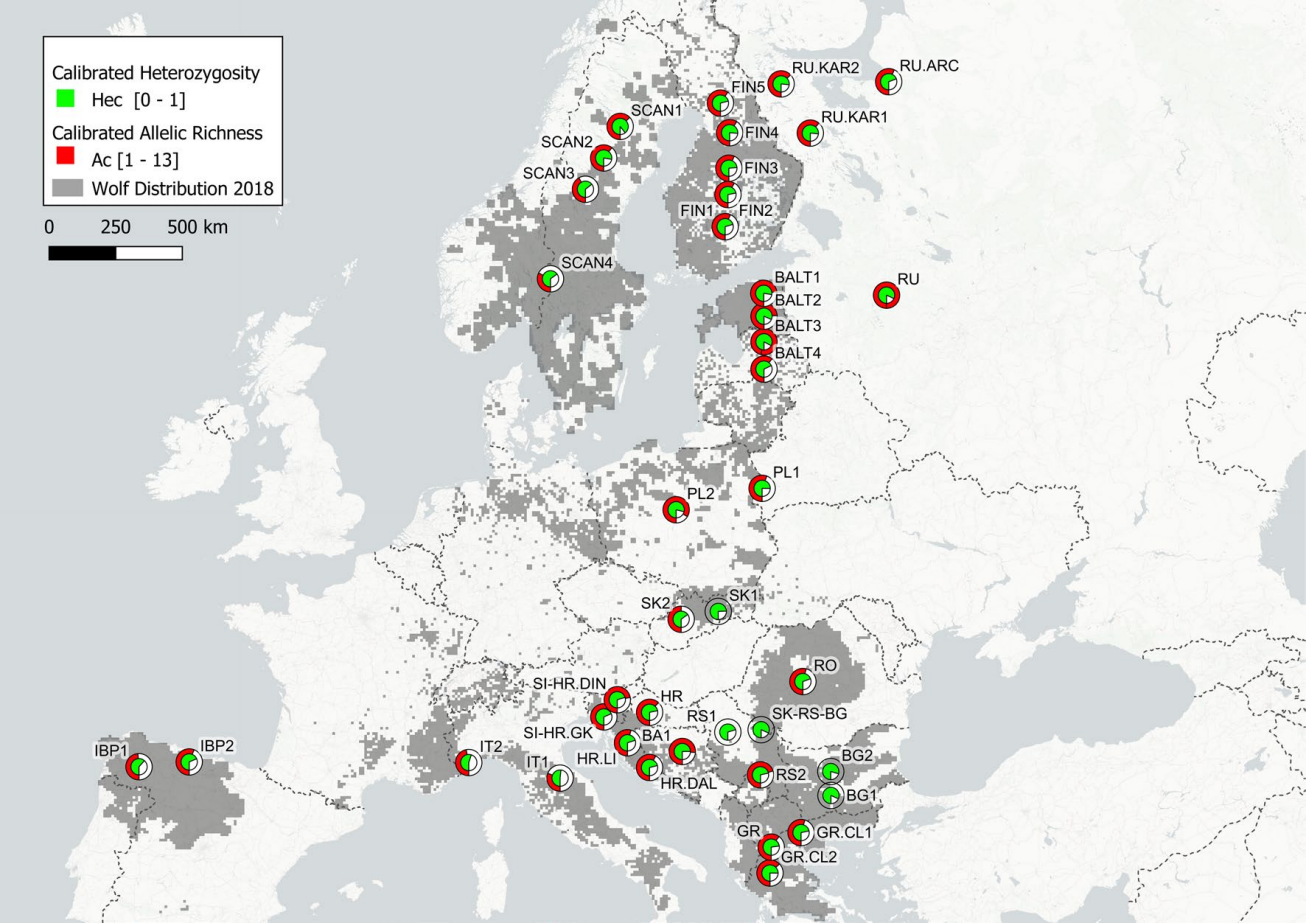
Distribution of wolves in Europe (based on Kaczensky et al.^26^) and genetic diversity indices reported in various studies calibrated using the “yardstick” method^11^. Overall, both calibrated heterozygosity and calibrated allelic richness showed a general increase from south-west to north-east, similar to that observed by Hindrikson et al.^15^. Studies that reported parameter values are included—see Supplemental Table S2.

Heterozygosity and allelic richness were generally higher in populations in N and NE Europe (European Russia, the Baltic States, Finland) (Fig. 3). In many cases *He* and *Ho* seem far apart (Fig. 1), suggesting a departure from HW equilibrium and possible population-level processes driving this departure. Examples are Iberia^45,46,49^, Poland^17^, and Bulgaria^24^, where the authors had noted known or probable underlying genetic structure. In general, allelic richness ratio and heterozygosity were lowest for Scandinavian wolves in 1991–2001 (*Art* = 0.685) and highest for wolves in European Russia (*Art* = 2.252).

Calibrated heterozygosity and allelic richness in Dinaric wolves were higher than those found in Scandinavian, Italian and Iberian wolves. In Carpathian wolves, both parameters were higher in Romania than in Slovakia, and comparable to those of Dinaric wolves. Our results from the Dinaric and Carpathian regions showed lower values for heterozygosity and allelic richness than those observed in NE Europe.

## Discussion

We assessed the genetic diversity of wolves in the Dinaric and Carpathian regions in Europe and examined our findings in a wider context at the continental level. The yardstick method^11^ allowed us to compare our results with genetic diversity parameters published in various studies from across Europe, which included samples originating from 1829 to 2018 (Supplemental Table S2). For Dinaric and Carpathian wolves we observed comparable calibrated heterozygosity and allelic richness, and genetic diversity values were, as expected, lower than in the large populations of northeastern Europe (Ref.^15^ and Supplemental Table S2). Within the Carpathians, the larger population segment in Romania also exhibited higher values than wolves in Slovakia. The meta-analysis provided a valuable continental-level overview of patterns of genetic diversity of this species, enabling a better understanding of the conservation status and ongoing recovery process of wolves in Europe.

### Meta-analysis of genetic diversity in European wolves

The observed distribution of genetic diversity in European wolves fits well with the general picture found in earlier studies^15,38,39,44,46,50–52^. Interestingly, none of the populations (Supplemental Table S2) showed exceedingly high or low heterozygosity despite some small populations have been isolated. This contrasts with the situation found in brown bears where major differences in heterozygosity were observed^11^. On the other hand, the differences in calibrated allelic richness (*Ac*) among wolf populations are considerable and, in many cases, correspond better with known population histories than the heterozygosity estimates. Ref.^15^ noted that allelic richness was distributed relatively evenly across Europe, showing only a weak spatial signal. In our study, *Ac* nonetheless shows a general increase from south-west to north-east, which corresponds with the distribution of genetic diversity across European populations observed by Ref.^15^. As allelic richness seems to be a good indicator of severe, short bottlenecks^53^, and may more effectively reflect a population’s long-term evolutionary potential^12,54^, temporal data on allelic richness can provide an effective means of monitoring smaller and relatively isolated populations identified as vulnerable (Ref.^15^ and references therein).

There is a general pattern of deviations from HW equilibrium in most reported wolf studies (Fig. 1). In some cases, this is clearly caused by the Wahlund effect^55^, but deviations occur at a relatively small spatial scale for a highly mobile mammal^29,30,49,56^. A simple explanation is that because many populations survived the persecution of previous centuries in very small numbers, high genetic drift caused by low effective population sizes leads to detectable genetic differentiation between populations, or even between population fragments within the same metapopulation, also at relatively small spatial scales. Founder effects during recolonization have resulted in persistent genetic drift^57^. Moreover, some areas were colonized from populations that were quite recently established by a limited number of founders, such as wolves in Denmark^58^, and a “double” founder effect could thus be affecting wolves in such areas.

However, in other cases there are clear deviations from HW equilibrium where a hidden substructure would be unexpected given the spatial and landscape characteristics of the area^59^. Such deviations seem more frequent in populations that are geographically close to other genetically differentiated wolf populations, than in divergent populations separated by larger geographic distances, as observed in our study. This general pattern of deviations from HW equilibrium at the continental scale may suggest that, as wolves in Europe continue to recover, gene flow may be starting between previously isolated populations. However, the detectable effects of gene flow usually follow clear and predictable patterns. At first, direct immigrants (i.e., dispersers) are expected to cause the Wahlund effect^55^ resulting in excess homozygotes and a positive *Fis*^60^*,* which may be misinterpreted as a sign of assortative breeding if no other analysis is done. This can be an explanation for populations that are above the diagonal dashed line in Fig. 1 where there is no reasonable expectation of genetic substructure (which would cause the same effect). Subsequent reproduction of these dispersers with resident wolves would cause an excess of heterozygotes (“isolate breaking”^55^), shifting the deviation from HW equilibrium in the other direction and causing negative *Fis*, which can also be misinterpreted as a sign of inbreeding avoidance. This may be the case for the populations below the dashed diagonal line in Fig. 1. Whereas these interesting processes require further research at the continental level and need to be interpreted using other knowledge and data about specific populations, an increase of gene flow could be seen as a positive development from the conservation perspective.

### Utility of the yardstick method for analyses of European wolves

Originally, the yardstick method calculates the ratios between the genetic diversity indices in target versus reference populations, but they are not always intuitive to interpret. We extended the method to calculate calibrated heterozygosity and allelic richness, which enables a much more intuitive interpretation. Moreover, we compared the “original” genetic diversity values obtained in the reviewed studies with the calibrated values (Fig. 2). The differences were small in studies with large sample sizes and large number of loci but became pronounced in small studies with a limited number of loci (Table S2). In many cases it would be impossible to meaningfully compare these studies with other studies of the same species. Using the yardstick method such comparisons became possible, but the confidence intervals around such estimates were quite large.

If sample size is small, estimates of some parameters, particularly allelic diversity, can become severely biased, and Ref.^11^ used bootstrap resampling to account for this issue when demonstrating the yardstick method. In our study the sample size for the reference (NW Dinaric) population was lower than in nine of the populations used for comparison. These nine studies all had relatively large sample sizes, and we therefore compared the published He and Ho for the shared loci directly (i.e., without resampling).

One challenge for our investigation was the availability of data from the published studies. In some cases, we were unable to calculate the calibrated allelic richness or observed heterozygosity ratio with the yardstick method for some of the reported population clusters, because the authors did not report locus-level allelic diversity for each separate unit. Neither were the temporal extents, sampling periods, or sampling areas clearly defined in all cases, and the results should therefore be interpreted with caution. For example, we noticed quite a difference between the genetic diversity observed in Slovakia in our study (study SK2) and that of Ref.^24^ (study SK1). Our sampling area was focused on northern Slovakia, and we included only 20 individuals, whereas Ref.^24^ had a sample of 194.

We genotyped 33 microsatellites, and only Ref.^45^ among the compared studies reported analyzing more markers (42 loci). However, the numbers of overlapping markers available for comparison between the reference and other populations varied widely, ranging from 21 to only four loci. The marker panels differed considerably among studies, reflecting the high diversity of markers available for canids, and nearly every study included here had its own marker panel. Conversely, in brown bears, where the number of markers routinely investigated is smaller, more than half of the compared studies had eight loci available for comparison with the reference population^11^. Whereas the high diversity of canid markers presents some challenges and complicates the comparison of populations that have few overlapping markers with the reference population, it also highlights the yardstick method’s utility in our objective of comparing wolves across Europe.

The yardstick method can inform conservation management for wide-ranging species where populations encompass multiple jurisdictions, such as large carnivores in the Carpathian and Dinaric-Balkan regions where populations of wolves, bears, and lynx (*Lynx lynx*) extend across multiple countries (Ref.^2,61^). The same holds for many ungulate species, such as red deer (*Cervus elaphus*) and wild boar (*Sus scrofa*) that are broadly distributed across Europe^62,63^. As with wolves in parts of our study area^64^, red deer, wild boar and other ungulate species in Europe have been affected by hybridization in parts of their range (reviewed in Ref.^4^). Such events confound analyses of genetic structure and variability, and make it difficult to distinguish natural patterns of gene flow and diversity from those influenced by human activities^62^. Broad-scale investigations that permit direct comparison of results across geographic regions therefore remain an important priority^4^.

### Genetic diversity of wolves in the Dinaric and Carpathian regions

The genetic diversity of wolves in the Dinaric Mountains reflects the population’s demographic history. Although it is comparable to many other recovering wolf populations in Europe, even the largest of these populations share the same history of persecution and recent recovery^20^ and may also have suffered population fragmentation and reduction of genetic diversity. Observed deviations from HW equilibrium are not unexpected and are probably due to the Wahlund effect as the population seems to be divided into two or three subpopulations^29^. Substructure at this scale may be a legacy of past persecution when wolves often survived in small and isolated population fragments^29,51,65^, where genetic drift quickly created a strong signal of population structure. Even relatively large populations, including Dinaric wolves, have therefore lost a considerable part of the original genetic diversity within each isolated fragment. There are indications, however, that the current recovery is starting to create gene flow between some population fragments^19,29,30,51,66,67^, which should help dissolve or at least reduce some of the existing population structure and augment genetic diversity.

Within the Carpathian Mountains, wolves in Romania seem to have higher genetic diversity than wolves in Slovakia, which is expected as they represent a major part (70%) of the Carpathian population^61^. Romanian wolves also appear relatively well-connected with wolves in the Carpathian Mountains of western Ukraine^23^. Wolves in Slovakia seem to have lower genetic diversity than their Dinaric counterparts, but still higher than the Scandinavian (study SCAN2) and Italian (study IT2) populations that are known to have gone through severe bottlenecks. Recent findings also suggest a dynamic situation with signs of recent gene flow between the Carpathian Mountains and wolves farther north and west^19,67^, which requires further monitoring and investigation.

### Implications for conservation and management

Although we observed considerable differences among wolf populations in Europe, even at finer spatial scales, these differences may gradually dissolve as recovering wolf populations become increasingly reconnected. This process is already seen in the Apennine Mountains^29^, in the Alps and Dinaric Mountains^29,30^, in this study, and possibly in the Carpathians Mountains^19^, and Poland^67^. Hence, this may be a wider phenomenon connected with the ongoing continent-wide recovery of wolves^20^.

The newly presented measures for calibrated heterozygosity and allelic richness allow a direct comparison of genetic diversity values among populations and a more intuitive interpretation of the results. However, it is still crucial to interpret the results carefully, including deviations from HW equilibrium, as this may indicate the presence of cryptic population structure^59^. For certain populations showing structure across relatively short geographic distances, natural selection has also been suggested as a possible contributing factor^18,30,51,68^ although it is difficult to see how this could have occurred in recent times. Given that most of these populations have been considerably reduced and their effective population size was (and probably still is) low, selection would need to be extremely strong not to be overpowered by genetic drift^69^, making adaptive evolutionary changes in such populations unlikely. However, this selection could have occurred in the past, in which case some of the currently observed population structure in wolves might not be a result of human activity, but possibly a result of natural selection (e.g., Ref.^70,71^). These issues have consequences for applied conservation of wild species, for example, whether some populations should be conserved in situ and admixture with other populations encouraged only via natural dispersal. These questions require further genomic research that can evaluate both neutral and functional genetic variation.

Gene flow from populations adapted to warmer and drier conditions could become increasingly important given rapid environmental changes^72^, and it would therefore seem farsighted to preserve the genetic diversity currently found in habitats where organisms are more likely to have experienced such selective pressures. The role of adaptive potential at range edges is important also for highly mobile species^73^, and future research could help clarify the extent to which allelic richness may function as an indicator of adaptive potential, where the yardstick method could be informative.

Much conservation effort is focused at the species level, including the backbone of European biodiversity conservation policy, the Habitats Directive (Council Directive 92/43/EEC on the conservation of natural habitats and of wild fauna and flora). Extinction rates for populations, however, are estimated to be three to eight times higher than those for species^74^. On the other hand, practical conservation in Europe is frequently done at the level of administrative units—countries, or even provinces—causing further “administrative fragmentation” of conservation management. To promote conservation, restore genetic diversity, and facilitate increased gene flow in wolves and other large carnivores, conservation managers should move towards population-level actions^75^. This process should be supported by the scientific community by shifting the focus from national-level research towards broader studies coordinated at the level of biogeographic regions (e.g., https://www.lifewolfalps.eu/) or even the entire continent. For highly mobile species such as large carnivores, broad-scale approaches likely present the best option to reduce human-wildlife conflicts and ensure their sustainable future in the human-dominated landscapes of modern-day Europe. Tracking allelic richness across the spatial scales relevant for gene flow in wolves and other highly mobile mammals could thus provide a cost-effective measure for genetic monitoring, especially given its capacity to connect past and present data across multiple jurisdictions.

### Supplementary Information

Supplementary Information 1.

Supplementary Tables.

## Data Availability

Most of the data on wolf genetic diversity that we refer to in our analysis come from previously published studies, cited in Supplemental Table S2 and Supplemental Note S3. The new data we have presented from Dinaric and Carpathian wolves are available in the Repository of the University of Ljubljana (https://repozitorij.uni-lj.si/IzpisGradiva.php?id=148315).
