# Supplementary material for: Wolf genetic diversity compared across Europe using the yardstick method
Source: Sci Rep. 2023 Aug 22;13:13727. doi: 10.1038/s41598-023-40834-x (PMC10444868; doi:10.1038/s41598-023-40834-x)
Supplement: Supplementary file 1 — Supplementary Information 1. [file 41598_2023_40834_MOESM1_ESM.docx]

**Supplemental Materials for “****Wolf genetic diversity compared across Europe using the yardstick method”**

**Supplemental Note S1. Materials and methods**

*Sample collection, storage, and DNA extraction*

For the Dinaric population we analyzed routinely collected tissue samples from dead wolves of known mortality, samples from individuals captured in telemetry studies and pups found in dens between 2003 and 2013 in Slovenia and Croatia, and reference samples from more than 1.600 noninvasive samples collected during the systematic genetic monitoring of wolves in the NW Dinaric Mountains (Slovenia and northern part of Gorski kotar in Croatia). In Croatia sampling was mainly opportunistic with lower intensity. No animal was killed or captured for the purpose of this study. Individuals were grouped into three subpopulations, NW Dinaric, Lika and Dalmatia, based on the location of sampling and previously genetic structure detected by Ref.^1^.

In Slovakia, a pilot study between December 2013 and June 2014 allowed the collection of 112 noninvasive samples, where 20 individuals were identified and used to estimate the genetic diversity of this population (Ref.^2^, unpublished data).

In Romania, a total of 254 noninvasive samples were collected during the winters 2017, 2018 and 2019. In the end 32 individuals were identified and used to estimate the genetic diversity of this population.


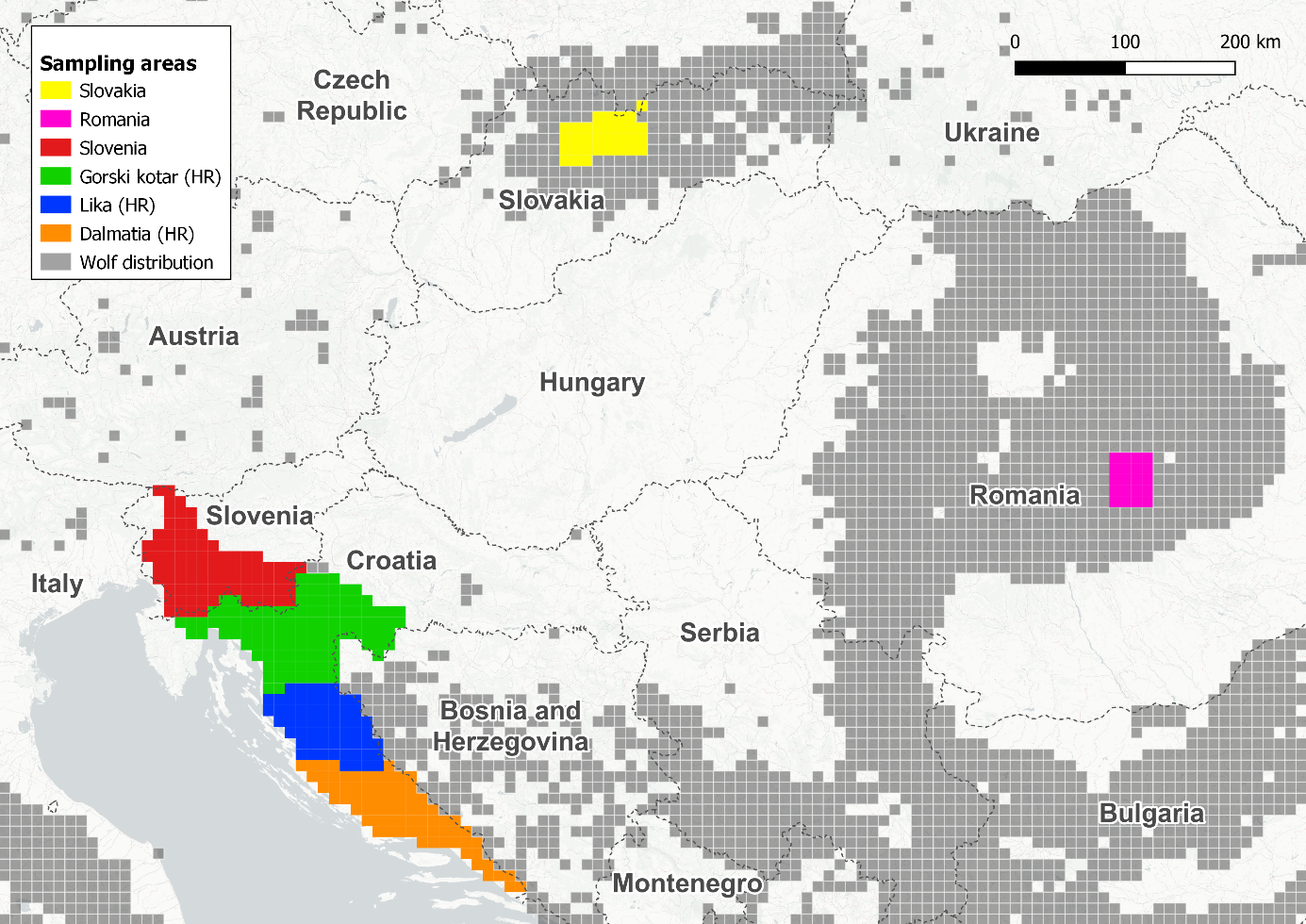


Figure S1: Sampling areas in the Dinaric Mountains (Slovenia and Croatia) and the Carpathian Mountains (Slovakia and Romania).

Most of the Dinaric wolf samples were tissues, collected in 96 % non-denatured ethanol and stored in 50 mL screw-cap tubes at -20 ˚C. Hair samples were collected in paper envelopes, air-dried and stored at room temperature in sealed bags with desiccant (silica gel). DNA extractions of both hair and tissue were carried out using GeneElute Mammalian Genomic DNA Miniprep Kit (Sigma-Aldrich, St Louis, MO, USA) following the manufacturer's instructions.

Scat samples were stored in 50 mL screw-cap tubes prefilled with 96% non-denatured ethanol or DETs buffer (in the case of Slovak samples). Urine samples were collected during snow tracking in winter. Urine-soaked snow (~ 30 mL) was collected into 50 mL screw-cap tubes prefilled with 15 mL of 96 % non-denatured ethanol and 3.2 mL of 0.5M EDTA and mixed thoroughly. For DNA extraction from scat and urine samples, we used Qiagen QIAmp^TM^ DNA Stool Mini Kit (Qiagen). Scats were prepared as described in Ref.^3^. Urine samples were extracted in a two-step process. First, they were thawed, mixed, and set aside for ~ 10 minutes for EDTA to sediment. Supernatant was removed and added to a 50 mL tube with 10 mL of 96 % non-denatured ethanol and 1.5 mL of 3M sodium acetate (pH 5.2). Distilled water was added to bring the total volume to 45 mL. After an overnight incubation at –20 °C, DNA was pelleted by cold centrifugation (8500g, 30 min, 6°C)^4,5^. In the second step, the pelleted material was transferred to a 2 mL tube and the extraction continued using the standard Qiagen kit protocol, with modifications described in Ref.^3^.

At reported livestock damages and opportunistically found natural prey remains, damage inspectors of the Slovenian Forest Service collected saliva samples. They were taken with cotton swabs from the area surrounding bite wounds, avoiding the blood of the prey as much as possible^6^. Most of the samples were collected at damage cases estimated to be one to two (maximum six) days old. Before swabbing, swabs were dipped in 96 % non-denatured alcohol to aid collection of oral mucosa cells attached to skin fat. We used dedicated forensic swabs that are stored in tubes containing desiccant, which ensures complete and rapid drying of samples (forensiX DNA sample storage tubes, Prionics). We extracted DNA using the QIAmp DNA Investigator Kit (Qiagen) following the manufacturer's instructions, with minor modifications. Swabs were left in the ATL buffer (part of the kit) in a heater-shaker at 56 °C overnight, followed by additional homogenization of the lysate using Qiashredder columns (Qiagen). Incubation at 70 °C took 20 minutes, each vortexing step took one minute, centrifugation steps at 8000 rpm were extended for one minute and centrifugation to completely dry the column membrane was performed at 12 000 rpm for 6 minutes.

Non-invasive samples from Romania were collected in DETs buffer (5 ml buffer in 8 ml vial). Once in the laboratory, they were stored at -20°C. The first step of processing the sample was treating it with S.T.A.R. buffer (Stool transport and recovery buffer, Roche) in ratio 1:1(400 µl buffer: 400 µl sample). Extractions were carried out on a Hamilton STARlet pipetting robot using Thermo Fisher Scientific MagMAX DNA Multi-Sample Kit.

*Genotyping and error checking*

For Dinaric wolves, we analyzed a total of 39 microsatellite markers and two sex specific markers (SRY and amelogenin) amplified in four PCR multiplexes. Five loci (Cxx.103, FH2079, FH2145, AHTh171 and INU005) were excluded from the downstream analysis due to amplification or genotyping problems, bringing the total number of usable microsatellite markers to 34, and two sex-ID loci to double-check designation of sex. For Carpathian wolves from Slovakia and Romania, we used a subset of 21 microsatellite markers. Markers, dyes, and primer concentrations are detailed in Supplemental Table S3. All PCRs were done using Qiagen Multiplex PCR kit. We prepared 10 µL reactions: 5 µL of Qiagen Mastermix, 1 µL of Q solution, 1 µL of template DNA of tissue samples and 2 µL of noninvasive samples and 1 µL of primers and water to obtain the appropriate concentration.

Fragment analysis was performed on an ABI 3130xl Genetic Analyzer (Applied Biosystems, USA). A mixture of 1 µL of the PCR product, 0.15 - 0.25 µL of GS500LIZ size standard (Applied Biosystems, USA) and 8.75 - 8.85 µL of formamide was loaded on the sequencer. We analyzed the output with GeneMapper software (version 4.0, Applied Biosystems, USA). We used the genotyping and quality assurance protocol described in Ref.^3^ to assure reliability of genotyping results. To estimate genotyping error and success rates, we grouped samples in a “low quality DNA group” – urine, scat, saliva, hair, decomposing tissue, and another “good quality DNA group” – tissue, saliva taken directly from captured animals, and blood. All noninvasive samples were genotyped at least two and up to eight times, and all tissue samples with unclear genotype at any locus were repeated for the entire multiplex containing that locus. Consequently, the genotyping error rates were estimated based on a considerably higher proportion of sample repeats than the 10 % suggested by Ref.^7^. For estimation of allelic dropout and false allele frequencies, we used the methods recommended by Ref.^8^. We calculated the quality index for each sample using the method proposed by Ref.^9^ and checked for the presence of null alleles with the program Micro-Checker 2.2.3 (Ref.^10^).

*Processing the samples*

All sampling materials (flasks, bags with individual swabs) had data-entry labels to keep the data with the sample. When received into the laboratory, each sample was recorded in a relational database and stored until processing. 2D barcodes were used to track samples from arrival in the laboratory and throughout the genotyping process to eliminate manual data entry errors and sample mix-ups. Each critical step in the analysis was either photo-documented, or the entire arrangement of samples automatically scanned using barcodes. For DNA extractions and PCR setup from noninvasive material we used a dedicated laboratory where we enforce rigorous contamination prevention rules for decontamination and cleaning, movement of personnel, equipment, and material. Pipette tips with aerosol barriers and negative controls were used throughout the extraction and PCR set-up.

*Hybridization analysis and identification of dogs, hybrids, and wolves*

To remove dog genotypes (samples inadvertently collected during noninvasive sampling) and putative wolf-dog hybrid genotypes from the dataset, we performed assignment tests using two methods – Principal Component Analysis (PCA) and the Bayesian clustering approach implemented in the program STRUCTURE V.2.3 (Ref.^11^). To detect and remove any possible wolf-dog hybrids, we used 52 samples of dogs as a reference for differentiation between wolves and dogs. Dogs were sampled by veterinarians during mandatory rabies vaccinations in villages within the wolf range in Slovenia. For Romanian wolves we also ran STRUCTURE with 22 dog samples collected in Romania. We targeted larger breeds and mongrels (crossbreeds) of appropriate (wolf-like) size. To identify wolf reference samples, we first analyzed all wolf and dog genotypes in a PCA using the R package Adegenet (Ref.^12^). As wolf reference genotypes we used individuals without any signs of hybridization, which were selected from our dataset using two criteria: 1) genotypes originating from Croatia, to avoid using data of closely related wolves sampled during intensive sampling in Slovenia, and 2) genotypes positioned far from the reference dog samples on the principal component separating the species (the median of the component with the highest eigenvalue was used as a threshold). We performed hybridization simulation between dogs (n = 52) and selected reference wolves (n = 105) using the program HYBRIDLAB (Ref.^13^), and ran Structure analysis (BURN IN = 100 000, MCMC = 1 000 000, n = 10, K = 2) to obtain threshold values for identification of F1, F2, wolf backcrosses and dog backcrosses, similar to Ref.^14^. To obtain permutated *p* values we used CLUMPP software (Ref.^15^) (*fullsearch* algorithm). All putative hybrid and dog genotypes were removed from further analysis, yielding 288 wolf genotypes considered to have no dog ancestry, which were used in downstream analyses.

**Supplemental Note S2. Results for Dinaric wolves**

*Genotyping, genotyping error rates and null alleles*

Five loci (Cxx.103, FH2079, FH2145, AHTh171 and INU005) were excluded from the downstream analysis due to amplification or genotyping problems, bringing the total number of usable microsatellite markers to 34, and two sex-ID loci to double-check designation of sex. Locus FH2004 had null alleles in the Dinaric population and was excluded from analysis. A total of 34 autosomal loci were analyzed for 331 putative wolves. From the downstream analysis we removed one dog genotype and 36 genotypes that indicated some level of genetic admixture of wolves and dogs. The gray wolf data set for the Dinaric population included 294 multilocus genotypes of individual wolves (288 and additional six individuals that were sampled outside the regular wolf range, but still clearly clustering with the Dinaric population).

On average, each sample was genotyped 2.3 times, but average number of amplifications per sample differs between samples of low DNA quality (3.9 times), and samples of good DNA quality (1.8 times). There was 0.77 % of missing data in the final dataset. Median allelic dropout rate in a single PCR was 4.1 % (0.85 % - 11.96 %, min - max) for lower DNA quality samples and 0.27 % (0 % - 2.17 %) for good quality samples. We detected false alleles only in three sample types (blood, direct saliva and decomposed tissues) at loci AHTh260, FH2010, INRA21, INU055 and REN162C04, CPH22 and INU030, and the overall rate of false alleles was 0.02 % per PCR. Because genotyping of each sample was repeated many times (in accordance with the sample’s quality), we consider the remaining errors in the dataset to be negligible for the purposes of this study.

**Literature cited**

1. Fabbri E, Caniglia R, Kusak J *et al.* (2014) Genetic structure of expanding wolf (Canis lupus) populations in Italy and Croatia, and the early steps of the recolonization of the Eastern Alps. *Mammalian Biology*, **79**, 138–148.

2. Rigg R, Skrbinšek T and Linnell J (2014). Engaging hunters and other stakeholders in a pilot study of wolves in Slovakia using non-invasive genetic sampling. Report to DG Environment, European Commission, Bruxelles. Contract no. 07.0307/2013/654446/SER/B.

3. Skrbinšek T, Jelenčič M, Waits L, Kos I, Trontelj P (2010) Highly efficient multiplex PCR of noninvasive DNA does not require pre-amplification. *Molecular Ecology Resources*, **10**, 495–501.

4. Hedmark E, Flagstad Ø, Segerstro P, Persson J, Landa A, Ellegren H. 2004. DNA-based individual and sex identification from wolverine (Gulo gulo) faeces and urine. Conservation Genetics, **5**: 405-410.

5. Hausknecht R., Gula R., Pirga B., Kuehn R. 2007. Urine - A source for noninvasive genetic monitoring in wildlife. Molecular Ecology Notes, **7, 2**: 208–212.

6. Sundqvist A.-K., Ellegren H., Vilà C. 2008. Wolf or dog? Genetic identification of predators from saliva collected around bite wounds on prey. Conservation Genetics, **9, 5**: 1275–1279.

7. Pompanon F, Bonin A, Bellemain E, Taberlet P (2005) Genotyping errors: causes, consequences and solutions. *Nature reviews. Genetics*, **6**, 847–859.

8. Broquet T, Petit E (2004) Quantifying genotyping errors in noninvasive population genetics. *Molecular ecology*, **13**, 3601–3608.

9. Miquel C., Bellemain E., Poillot C., Bessière J., Durand A., Taberlet P. 2006. Quality indexes to assess the reliability of genotypes in studies using noninvasive sampling and multiple-tube approach. Molecular Ecology Notes, 6, 4: 985–988.

10. Van Oosterhout C., Weetman D., Hutchinson W.F. 2006. Estimation and adjustment of microsatellite null alleles in nonequilibrium populations. Molecular Ecology Notes, 6, 1: 255–256

11. Pritchard JK, Wen X, Falush D (2010) *Documentation for structure software: Version 2.3*. University of Chicago, Chicago.

12. Jombart T (2008) Adegenet: a R package for the multivariate analysis of genetic markers. *Bioinformatics*, **24**, 1403–1405.

13. Nielsen EE, Bach LA, Kotlicki P (2006) HYBRIDLAB (version 1.0): A program for generating simulated hybrids from population samples. Molecular Ecology Notes, 6, 971–973.

14. Godinho R, Llaneza L, Blanco JC *et al.* (2011) Genetic evidence for multiple events of hybridization between wolves and domestic dogs in the Iberian Peninsula. *Molecular Ecology*, **20**, 5154–5166.

15. Jakobsson M, Rosenberg NA (2007) CLUMPP: a cluster matching and permutation program for dealing with label switching and multimodality in analysis of population structure. *Bioinformatics*, **23**, 1801–1806.

**Supplemental Note S3.** Complete bibliographic details for the studies shown in Supplemental Table S2 and used for the comparison of populations.

Aspi J., Roininen E., Ruokonen M., Kojola I., Vilà C. 2006. Genetic diversity, population structure, effective population size and demographic history of the Finnish wolf population. Molecular Ecology, 15, 6: 1561–1576

Aspi J., Roininen E., Kiiskilä J., Ruokonen M., Kojola I., Bljudnik L., Danilov P., Heikkinen S., Pulliainen E. 2009. Genetic structure of the northwestern Russian wolf populations and gene flow between Russia and Finland. Conservation Genetics, 10, 4: 815–826

Bakan, J., Lavadinovć, V., Popović, Z. & Paule, L. (2014). Genetic differentiation of grey wolf population (Canis lupus L.) from Balkan and Carpathians. Balkan Journal of Wildlife Research 1, 87–93.

Bensch S., Andrén H., Hansson B., Pedersen H.C., Sand H., Sejberg D., Wabakken P., Akesson M., Liberg O. 2006. Selection for heterozygosity gives hope to a wild population of inbred wolves. PloS One, 1, 1: 1–7.

Czarnomska S.D., Jędrzejewska B., Borowik T., Niedziałkowska M., Stronen A. V., Nowak S., Mysłajek R.W., Okarma H., Konopiński M., Pilot M., Śmietana W., Caniglia R., Fabbri E., Randi E., Pertoldi C., Jędrzejewski W. 2013. Concordant mitochondrial and microsatellite DNA structuring between Polish lowland and Carpathian Mountain wolves. Conservation Genetics, 14, 3: 573–588.

Djan M, Šnjegota D, Veličković N, Stefanović M, Obreht Vidaković D, Ćirović D. 2016. Genetic variability and population structure of grey wolf (*Canis lupus*) in Serbia. Russian Journal of Genetics 52: 821-827.

Fabbri E, Caniglia R, Kusak J *et al.* (2014) Genetic structure of expanding wolf (Canis lupus) populations in Italy and Croatia, and the early steps of the recolonization of the Eastern Alps. *Mammalian Biology*, **79**, 138–148.

Flagstad Ø., Walker C.W., Vilà C., Sundqvist A.-K., Fernholm B., Hufthammer A.K., Wiig Ø., Koyola I., Ellegren H. 2003. Two centuries of the Scandinavian wolf population: patterns of genetic variability and migration during an era of dramatic decline. Molecular Ecology, 12, 4: 869–880.

Godinho R, Llaneza L, Blanco JC *et al.* (2011) Genetic evidence for multiple events of hybridization between wolves and domestic dogs in the Iberian Peninsula. *Molecular Ecology*, **20**, 5154–5166.

Hindrikson M., Remm J., Männil P., Ozolins J., Tammeleht E., Saarma U. 2013. Spatial genetic analyses reveal cryptic population structure and migration patterns in a continuously harvested grey wolf (*Canis lupus*) population in North-Eastern Europe. PLoS ONE, 8, 9: e75765, doi:10.1371/ journal.pone.0075765: 12 str.

Jansson E., Ruokonen M., Kojola I., Aspi J. 2012. Rise and fall of a wolf population: Genetic diversity and structure during recovery, rapid expansion and drastic decline. Molecular Ecology, 21: 5178–5193

Jędrzejewski W., Branicki W., Veit C., MeĐugorac I., Pilot M., Bunevich A.N., Jędrzejewska B., Schmidt K., Theuerkauf J., Okarma H., Gula R., Szymura L., Förster M. 2005. Genetic diversity and relatedness within packs in an intensely hunted population of wolves *Canis lupus*. Acta Theriologica, 50, 1: 3–22.

Karamanlidis A. A, Sylwia D. Czarnomska S.D, Kopatz A, Georgiadis L, Jedrzejewska B. (2016) Wolf population genetics at the south-eastern edge of their European range. Mammalian Biology, **81,** 506-510.

Moura AE, Tsingarska E, Dabrowski MJ *et al.* (2013) Unregulated hunting and genetic recovery from a severe population decline: The cautionary case of Bulgarian wolves. *Conservation Genetics*, **15**, 405–417.

Randi E., Lucchini V. 2002. Detecting rare introgression of domestic dog genes into wild wolf (Canis lupus) populations by Bayesian admixture analyses of microsatellite variation. Conservation Genetics, 3: 31–45.

Rigg R, Skrbinšek T and Linnell J (2014). Engaging hunters and other stakeholders in a pilot study of wolves in Slovakia using non-invasive genetic sampling. Report to DG Environment, European Commission, Bruxelles. Contract no. 07.0307/2013/654446/SER/B.

Sastre N, Vilà C, Salinas M *et al.* (2010) Signatures of demographic bottlenecks in European wolf populations. *Conservation Genetics*, **12**, 701–712.

Šnjegota D, Stefanović M, Veličković N, Ćirović D, Djan M. 2018. Genetic characterization of grey wolves (*Canis lupus* L. 1758) from Bosnia and Herzegovina: implications for conservation. Conservation Genetics 19: 755-760.

**Supplemental Note S4.** Complete bibliographic details for the Loci with associated data used and our study shown in Supplemental Table S3.

Bellemain E., Taberlet P. 2004. Improved noninvasive genotyping method: application to brown bear (*Ursus arctos*) faeces. Molecular Ecology Notes, **4**, 3: 519–522.

Breen M., Jouquand S., Renier C., Mellersh C.S., Hitte C., Holmes N.G., Chéron A., Suter N., Vignaux F., Bristow A.E., Priat C., McCann E., André C., Boundy S., Gitsham P., Thomas R., Bridge W.L., Spriggs H.F., Ryder E.J., Curson A., Sampson J., Ostrander E.A., Binns M.M., Galibert F. 2001. Chromosome-specific single-locus FISH probes allow anchorage of an 1800-marker integrated radiation-hybrid/linkage map of the domestic dog genome to all chromosomes. Genome Research, **11, 10**: 1784–1795.

Dolf G., Schläpfer J., Gaillard C., Randi E., Lucchini V., Breitenmoser U., Stahlberger-Saitbekova N. 2000. Differentiation of the Italian wolf and the domestic dog based on microsatellite analysis. Genetics, Selection, Evolution, **32**: 533–541.

Francisco L. V, Langston A.A., Mellersh C.S., Neal C.L., Ostrander E.A. 1996. A class of highly polymorphic tetranucleotide repeats for canine genetic mapping. Mammalian Genome, **7**: 359–362.

Fredholm M., Winterø A.K. 1995. Variation of short tandem repeats within and between species belonging to the Canidae family. Mammalian Genome, **6**: 11–18.

Guyon R., Lorentzen T.D., Hitte C., Kim L., Cadieu E., Parker H.G., Quignon P., Lowe J.K., Renier C., Gelfenbeyn B., Vignaux F., DeFrance H.B., Gloux S., Mahairas G.G., Andre C., Galibert F., Ostrander E.A. 2003. A 1-Mb resolution radiation hybrid map of the canine genome. Proceedings of the National Academy of Sciences, **100, 9**: 5296–5301.

Holmes N.G., Dickens H.F., Parker H.L., Binns M.M., Mellersh C.S., Sampson J. 1995. Eighteen canine microsatellites. Animal Genetics, **26**: 132–133.

Mariat D., Kessler J.L., Vaiman D., Panthier J.J. 1996. Polymorphism characterization of five canine microsatellites. Animal Genetics, **27, 6**: 434–435.

Ostrander E.A., Sprague G.F., Rine J. 1993. Identification and characterization of dinucleotide repeat (CA)n markers for genetic mapping in dog. Genomics, **16**: 207–213.

Pedersen N.C., Liu, H., Greenfield D.L., Griffioen Echols L. 2012. Multiple autoimmune diseases syndrome in Italian greyhounds: Preliminary studies of genomewide diversity and possible associations within the dog leukocyte antigen (DLA) complex. Vet. Immunol. Immunopathol. **145**, 264–276.

Shibuya H., Collins B.K., Huang T.H., Johnson G.S. 1994. A polymorphic (AGGAAT)n tandem repeat in an intron of the canine von Willebrand factor gene. Animal Genetics, **25**, 2: 122.

Thomas R., Holmes N.G., Fischer P.E., Dickens H.F., Breen M., Sampson J., Binns M.M. 1997. Eight canine microsatellites. Animal Genetics, **28, 2**: 153–154.
